# Hysterectomy: Let’s Step Up the Ladder of Evidence to Look Over the Horizon

**DOI:** 10.3390/jcm11236940

**Published:** 2022-11-25

**Authors:** Andrea Giannini, Ottavia D’Oria, Giorgio Bogani, Violante Di Donato, Enrico Vizza, Vito Chiantera, Antonio Simone Laganà, Ludovico Muzii, Maria Giovanna Salerno, Donatella Caserta, Sandro Gerli, Alessandro Favilli

**Affiliations:** 1 Department of Medical and Surgical Sciences and Translational Medicine, PhD Course in “Translational Medicine and Oncology”, Sapienza University, 00185 Rome, Italy; 2 Department of Maternal and Child Health and Urological Sciences, Policlinico Umberto I, Sapienza University of Rome, 00185 Rome, Italy; 3 Obstetrics and Gynecological Unit, Department of Woman’s and Child’s Health, San Camillo-Forlanini Hospital, 00152 Rome, Italy; 4 Gynecologic Oncology Unit, Department of Experimental Clinical Oncology, IRCSS-Regina Elena National Cancer Unit Institute, 00144 Rome, Italy; 5 Unit of Gynecologic Oncology, Department of Health Promotion, Mother and Child Care, Internal Medicine and Medical Specialties (PROMISE), ARNAS “Civico-Di Cristina-Benfratelli”, University of Palermo, 90133 Palermo, Italy; 6 Department of Medical and Surgical Sciences and Translational Medicine, Gynecology Division, Sant’Andrea University Hospital, Sapienza University of Rome, Via di Grottarossa 1035, 00189 Rome, Italy; 7 Section of Obstetrics and Gynecology, Department of Medicine and Surgery, University of Perugia, 06123 Perugia, Italy

Hysterectomy is one of the most common non-obstetric gynecological surgical procedures carried out in Western countries [1]. In the United States, it is the second most common surgery after cesarean section, in reproductive-age women [1]. There are several gynecological recommendations for hysterectomy, including malignant and benign conditions. The most frequent indications of hysterectomy are leiomyomas, abnormal bleeding, pelvic floor prolapse and cancers [2,3,4]. Some of these indications are still debated [1,5]. In recent years, the rate of the surgical uterus removal has decreased due tothe development of screening and prevention programs, including ultrasonography, diagnostic hysteroscopy and the advancement of minimally invasive surgery techniques, such as endometrial ablation for abnormal uterine bleeding, vaginal and hysteroscopic myomectomy, and/or the use of hormonal therapies [5,6,7,8].

The surgical approach to performing hysterectomy include abdominal, vaginal, traditional laparoscopic, robot-assisted laparoscopic, single-port laparoscopic/robotic surgery, and vaginal natural orifice transluminal endoscopic surgery (vNOTES) [9,10]. The surgical approach to hysterectomy depends on the clinical indication and condition of the patient, the technical skills of the surgeon, and patient preference. Vaginal hysterectomy, if it is technically feasible, is preferred over an abdominal approach because of more rapid postoperative recovery. In recent years, minimally invasive surgery (MIS) has become more available and has become the gold standard for benign conditions. The American Association of Gynecologic Laparoscopists suggests that many hysterectomies for benign conditions should be carried out using laparoscopy, since accumulating evidence confirms that laparoscopic hysterectomy is associated with less perioperative morbidity compared to abdominal hysterectomy [11,12,13]. The robotic technique has a high diffusion among gynecologic surgeons in developed countries, causing a decrease in the use of conventional laparoscopy since the approval of the DaVinci surgical system (Intuitive Surgical Inc., Sunnyvale, CA, USA) by the United States Food and Drugs Administration (FDA) for gynecologic surgery in 2005 [14]. Other emerging surgical robots include the Hugo (produced by Medtronic) and Versius (produced by CMR) robotic platforms. This robotic approach has several specific surgical benefits: three-dimensional vision, quicker learning curve, high definition, greater precision, and greater ergonomic potential. The major limitations of robotic surgery are its costs and unavailability in all centers.

The use of MIS techniques has increased for the treatment of gynecological cancers too, particularly for early-stage cervical cancer (ECC) [15]. The utilization of MIS techniques is more beneficial in terms of blood loss, the rate of postoperative complications, length of hospitalization, and cosmetic results, making hysterectomy an intervention with less emotional impact, even in the case of cancer. After the results of the Laparoscopic Approach to Cervical Cancer (LACC) trial, different studies are currently underway to confirm the safety of MIS in these patients in terms of oncological outcomes. By comparing MIS with open radical hysterectomy in ECC, the LACC trial unexpectedly reported inferior oncological outcomes, showing a 6.6 times greater likelihood of death associated with MIS, and lower disease-free survival (DFS) and overall survival (OS) in the MIS group [16,17,18,19,20]. The recent Multicenter study of Minimally invasive surgery versus Open Radical hysterectomy (MEMORY) study denied these findings in the management of ECC, showing that MIS compared to open radical hysterectomy for cervical cancer did not appear to compromise oncologic outcomes, with similar DFS and OS [21], and these elements was further confirmed by a more recent data analysis [22]. The results of the Robot-assisted approach to cervical cancer (RACC) trial could change these findings, confirming the oncologic safety of robot-assisted surgery for early-stage cervical cancer as compared with standard laparotomy [23]. In addition, the oncological outcomes after laparoscopic and laparotomic hysterectomies are almost overlapping even when we refer to other malignancies, such as endometrioid endometrial cancer [24].

Despite the high diffusion of the minimally invasive approach, abdominal hysterectomy remains a very common surgical intervention. The knowledge of this surgical intervention is essential for new generations of gynecologists. The most common indications for the abdominal hysterectomy are large uterine size, previous abdominal surgeries, severe endometriosis and adhesions history. However, there are no specific indications for uterine weight or size and abdominal hysterectomy, and studies show that minimally invasive techniques, such as laparoscopy, can safely remove large uteri too [25,26].

There are also obstetric indications for abdominal hysterectomy, in most cases in an emergency regime: severe postpartum hemorrhage (PPH)/atony refractory to pharmacological treatments, placenta accrete spectrum disorders (PAS), hypertensive disorders, disseminated intravascular coagulation (DIC) [27]. Nonetheless, peripartum hysterectomy is burdened with a higher morbidity and mortality rate [28].

Hysterectomy is a surgical procedure with potential surgical complications, with the highest rate within 30 days: infectious consequences, hemorrhage, thromboembolic disease, urinary and/or intestinal tract injury, nerve injury and vaginal cuff dehiscence [18]. The complication rate is lower in patients treated with the minimally invasive technique [29,30]. Despite abdominal hysterectomy remains a routine surgery, and it is well known that this type of surgical approach is characterized by a long recovery time, high costs and a high rate of complications.

Hysterectomy can be a difficult choice for women, with sexual and psychological changes that may led to a non-negligible risk of post-traumatic stress [31]. Therefore, a patient who has to undergo this surgery must know all her options. What are the future perspectives for the best surgical outcomes? Laparoscopic and robotic approaches have partially answered this question, making the impact of this surgery less traumatic for patients in terms of hospital stay, recovery time, and aesthetic results. Considering improvement in patient outcomes and health care systems, MIS should be preferred when feasible and safe. Nevertheless, hysterectomy remains an interesting and challenging research field for surgical teams.
